# Mapping the Relationship of Contributing Factors for Preclinical Alzheimer’s Disease

**DOI:** 10.1038/srep11259

**Published:** 2015-07-20

**Authors:** Lin Shi, Lei Zhao, Adrian Wong, Defeng Wang, Vincent Mok

**Affiliations:** 1 Department of Medicine and Therapeutics, The Chinese University of Hong Kong, Shatin, NT, Hong Kong SAR; 2 Chow Yuk Ho Center of Innovative Technology for Medicine, The Chinese University of Hong Kong, Shatin, NT, Hong Kong SAR; 3 Department of Imaging and Interventional Radiology, The Chinese University of Hong Kong, Shatin, NT, Hong Kong SAR; 4 Research Center for Medical Image Computing, The Chinese University of Hong Kong, Shatin, NT, Hong Kong SAR

## Abstract

While detecting and validating correlations among the contributing factors to the preclinical phase of Alzheimer’s disease (pAD) has been a focus, a potent meta-analysis method to integrate current findings is essential. The entity-relationship diagram with nodes as entities and edges as relationships is a graphical representation that summarizes the relationships among multiple factors in an intuitive manner. Based on this concept, a new meta-analysis approach with this type of diagram is proposed to summarize research about contributing factors of pAD and their interactions. To utilize the information for enriched visualization, width and color of the edges are encoded with reporting times, number of pAD subjects, correlation coefficient, and study design (cross-sectional or longitudinal). The proposed *Probabilistic Entity-Relationship Diagram (PERD)* demonstrated its effectiveness in this research for studying pAD. Another kind of diagram with occurrence order for some factors was also proposed to provide sequential information of the factors. In addition, *PERD* could potentially develop into an online application named *PERD-online*, which would help researchers to pool findings on the same relationships and guide further tests to validate uncertain relationships in *PERD*. *PERD* as a generic graphical meta-analysis tool can also be applied in studying other multifactorial diseases.

Alzheimer’s Disease (AD) is the most common form of dementia which usually presents in patients above 65 years of age^1^. The disease starts with a preclinical phase, preclinical AD (pAD), in which AD neuropathology begins to accumulate but cognitive performance presents as normal^2^. The early detection of pAD has become increasingly important because early intervention has the promise to slow down the progression towards AD. While extensive research has been directed towards improving existing imaging methods and understanding the underlying mechanism of the disease, a holistic understanding of contributing factors in pAD and their relationships is still lacking. Besides further research on potential biomarkers of pAD, an effective visualized meta-analysis approach for better understanding the existing findings is needed.

Diagrams with nodes representing factors and edges representing their relationships have been used in biological research on multifactorial progresses. For instance, a metabolic network enables visualization of large-scale structures in the organization of various organisms^3^. With a higher translational value, the cardiovascular network modeled through reviews with relevant progresses^4^ elucidates some higher-order interactions underlying the clinical traits in cardiovascular disease. This type of network analysis is also promising in the study of other multifactorial diseases^5^.

A new graphical meta-analysis method, namely the *Probabilistic Entity-Relationship Diagram (PERD)*, is introduced in this paper. It is designed as a color map with nodes (biomarkers) and edges (correlations) showing the relationship of reported contributing factors resulting in pAD. This map will not only help researchers to find the biomarkers that are associated with pAD, but also provide inspirations to design trials for better understanding pAD pathology.

## Material and Methods

### Keywords of the survey

Preclinical AD; Imaging.

### Criteria

The literature concerning the study of pAD in the past five years was surveyed and only the papers that involved imaging for test were used for meta-analysis. However, if the known biomarkers were not reported to be directly related with pAD in the past five years, the time span for surveying the literature would be expanded to ten years or even more to provide a reasonable appearance for the graphical meta-analysis. To make sure the included studies were about pAD rather than MCI or AD, we screened the papers based on the participants involved in the research. Namely, the subjects were either observed in conversion from normal control to MCI/AD in longitudinal studies, or grouped by well-established pAD biomarkers (e.g. Aβ42 (CSF/PET)) when they did not suffer cognitive impairment in cross-sectional studies. In addition, as familial (early-onset) AD cases differed from sporadic (late-onset) cases in pathophysiology (e.g. Aβ42 (CSF) increases early in sporadic AD^6^, while it decreases early in familial AD^7^) , we excluded the studies that focused on familial pAD subjects. Finally, we only included the studies with significant findings to construct the *PERD*. The significance level for the p-value was defined as 0.1 rather than 0.05 to include more potential significant biomarkers that might be sacrificed by the small sample size of pAD subjects available, but their visual significance in *PERD* will be simultaneously controlled through graph generation.

### Database used

PubMed was the only database used for data collection and analysis. According to the aforementioned keywords and criteria of literature survey, 24 studies related to imaging of pAD were included, and all the collected studies were used for graphical meta-analysis. In particular, 22 studies that reported findings of overall correlations between pAD biomarkers were utilized to generate the major diagram of *PERD*, while the detailed regional findings from these studies were further visualized in a complementary diagram. An extra diagram was proposed to present the occurrence order of the biomarkers with the findings from 3 aforementioned studies and 2 additional studies that were not used in the major diagram.

### Generation of the Probabilistic Entity-Relationship Diagram of preclinical AD

The *PERD* of pAD was a colored diagram summarizing the imaging biomarkers of pAD and plotting their relationships. Well-established CSF pAD-predictors were also included. The nodes denoted the various contributors of pAD, while the edges stood for the corresponding relationship between the nodes. The width and color of the edge were designed to represent the strength of the relationship by integrating reporting times, number of subjects diagnosed with pAD, study design (longitudinal or cross-sectional), correlation coefficient and corresponding significance level (p-value) of the relationship. The colored map of relationships was generated with *Microsoft Visio 2013,* with the parameters specifying width and color of edges in Table 1. Although age is the most important predictor of AD, it was not included in visualization due to the inconsistency in statistical approaches for age. Instead, the age statistics reported in each literature were listed in Table 2.

#### Width of the Edge

The width of edge showed the degree of consensus on a certain correlation. Among the factors describing the relationship of biomarkers, reporting times (*T*) and the number of pAD subjects (*N*) indicated the scale of work on the agreement of the relationship, which governed edge width (*W*). In addition, weighting was used to reflect the study design (cross-sectional or longitudinal) and the size of relationship significance (*p*-*value*). Compared to cross-sectional studies, longitudinal studies provide more convincing results due to observation of conversion of normal subjects to pAD onset. Therefore, a penalty coefficient *α* (*α *< 1) was proposed to adjust the significance of the results from cross-sectional studies. In order to make best of the existing studies, the surveyed literatures were weighted by a p-value penalty (*β*), which was designed to enlarge the impact of the findings with high significant level (Table 1).


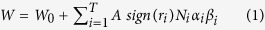


The equation (1) was applied to compute the width of the edge. *W*_0_ was the default width of edge for observed correlations (*W*_0_ = 0.5pt). The weight *A* was empirically designed to adjust the appearance of the edges. The combined term on the right contained the results of various reports on the same relationship, and the parameter *i* referred to the result of a single report. Definitions of other parameters were further explained in Table 1.

#### Color of the Edge

**Figure 1:**
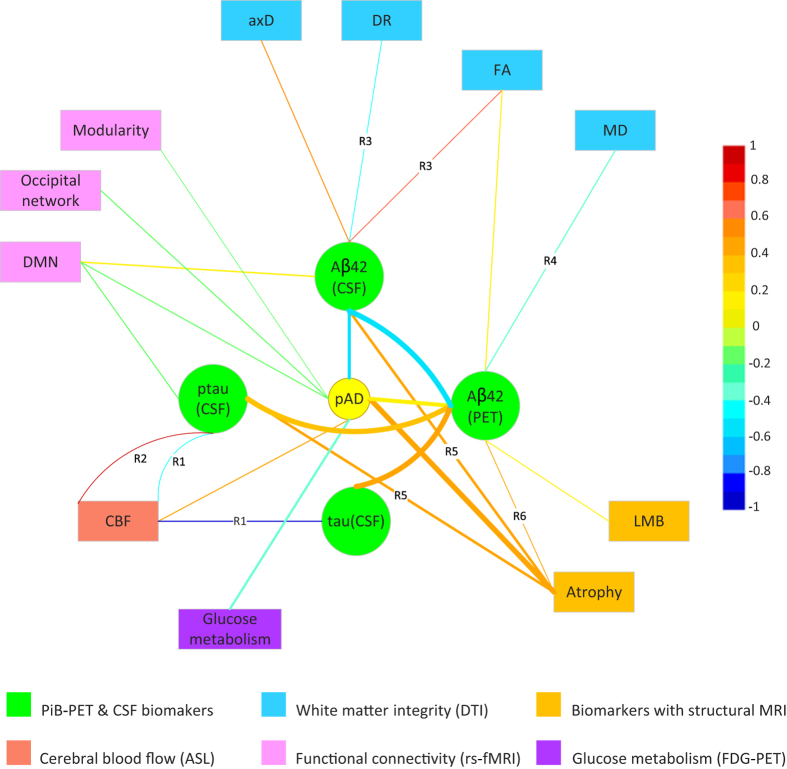
The Probabilistic Entity-Relationship Diagram (*PERD*) of preclinical AD. FA = Fractional Anisotropy; MD = mean diffusivity; DR = radial diffusivity; axD = axial diffusivity; LMB = lobar microbleed; CBF = cerebral blood flow; DMN = default mode network; R1 = right superior medial frontal lobe; R2 = left frontal-temporal lobe; R3 = left fornix; R4 = lateral frontal gray matter; R5 = cerebral cortex; R6 = basal forebrain.

**Figure 2:**
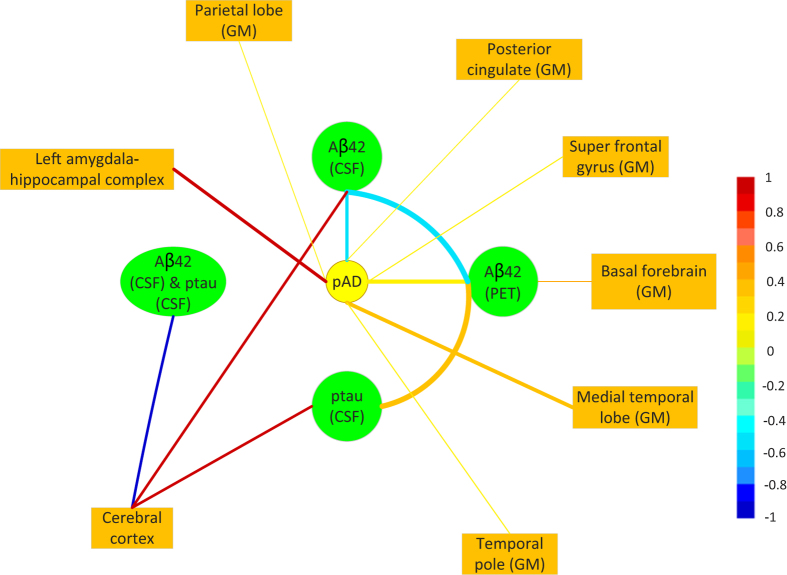
*PERD* of relationship between atrophy of different anatomical positions and pAD or its significant biomarkers. GM = gray matter.

Different colors represented the sign and strength of the correlation. The strength of correlation was illustrated with coded color from yellow to red for positive correlation and green to blue for negative correlation. Bearing in mind the difference of reports on the same relationship, we used weighted average correlation coefficient (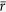) to designate the RGB value of the edge (*a lookup table was provided in color, as was shown [Disp-formula eq4]in the colorbar of* Fig. 1 and Fig. 2). This coefficient was composed of the reported correlation coefficient (*r*_*i*_), the number of pAD subjects, and the cross-sectional penalty (*α*_*i*_) (equation (2)). For those studies involving group comparisons or regression rather than correlation, *r*_*i*_ was estimated empirically based on the p-value and the sign of the correlation, where 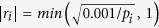 (*we defined 0.001 as a strict significance level*).
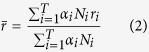


## Results

Figures 1,2 below show the relationship among possible pAD contributing factors based on the literature findings (Table 2). In general, studies on the relationship of contributors of pAD focus on some specific regions of the brain. To include both global and regional findings of the listed biomarkers, while keeping the visualization neat and clear, Fig. 1 is taken as the main diagram of *PERD* (all the biomarkers listed in Table 2 are shown). The regional findings with no more than three different regions are specifically labeled. In detail, the labeled edges in Fig. 1 indicate the correlations are between regional imaging biomarkers and pAD or well-established pAD biomarkers. The edges without labels correspond to correlations that involve biomarkers with global findings or specific regional findings (with more than three different regions (e.g. atrophy) (Fig. 1)). For the biomarkers with more than three regional findings, they are visualized in a complementary diagram (Fig. 2).

The color bar on the right relates the correlation coefficients to the connections of the nodes. A correlation coefficient of +1 will be reported by red, and a correlation coefficient of −1 by blue. Besides coding color for the edges, we also color-code the major findings of biomarkers (Fig. 1), which are classified through imaging methods. For example, white matter integrity examined by DTI is set to blue (including Fractional Anisotropy (FA), mean diffusivity (MD), radial diffusivity (RD) and axial diffusivity (axD)). Considering that Aβ42(CSF/PET) has been widely used to depict the status of pAD^8^ and that the studies reporting the correlation of Aβ42(CSF/PET) and pAD has become increasingly scarce recently (Table 2), we include the studies that reported correlation of imaging biomarkers and pAD in addition to compute the width of edge between Aβ42 (CSF/PET) and pAD. In this way, the diagram appears more reasonable with available data.

As is shown from Fig. 1, brain atrophy and glucose metabolism are the most significant biomarkers for pAD in addition to Aβ42 (CSF/PET) in terms of edge width. The possibility that subjects suffer from pAD rises greatly if they have more severe brain atrophy or lower glucose metabolism. In addition, CSF tau and p-tau are relatively significant contributors to pAD because of their strong connections to Aβ42 (PET), a well-established biomarker for pAD. As for the color of the edge, regional cerebral blood flow (in right superior medial frontal lobe (R1) and left frontal-temporal lobe (R2)) and regional FA (in left fornix (R3)) are significantly related to known biomarkers of pAD (PiB-PET and CSF biomarkers). The widths and colors of other edges do not differ obviously, probably because the studies on these biomarkers are still lacking and that the number of participants with pAD that involve corresponding biomarkers is very small.

In Fig. 2, atrophy, which is reported with the most detailed anatomical information, is independently visualized with regional findings. In this diagram, the atrophy of left amygdala-hippocampal complex and medial temporal lobe in gray matter are significantly correlated with pAD directly in terms of edge width, and the atrophy of left amygdala-hippocampal complex also has significant prediction effect in terms of color. In addition, the atrophy of cerebral cortex is significantly associated with pAD indirectly through well-established pAD contributors (e.g. Aβ42 (CSF)). An interesting finding from this graph is that the interaction of Aβ42 (CSF) and p-tau (CSF) produces an inverse effect compared to either of them when studied alone. Aβ42 (CSF) and p-tau (CSF) both have positive correlation with cerebral cortex volume when present alone, but the presence of them together will invert this relationship.

**Figure 3:**
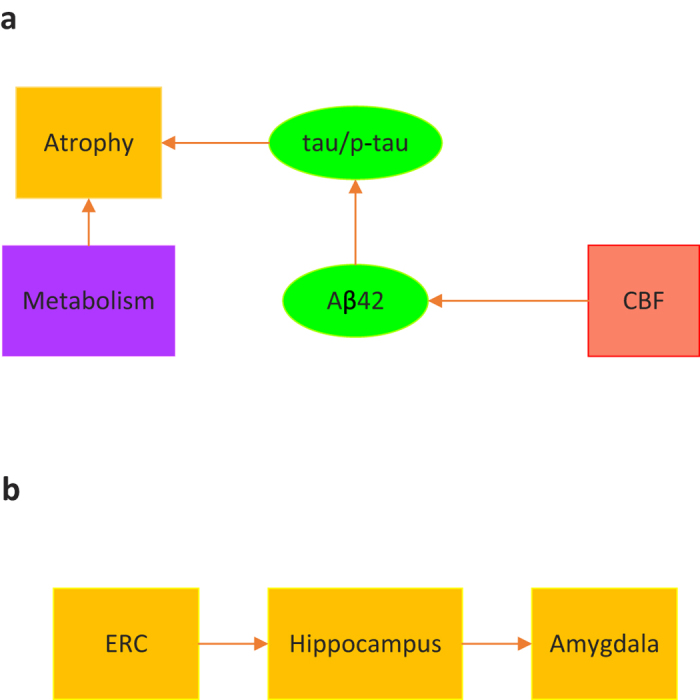
Occurrence of pAD-biomarkers in sequence proposed by the surveyed literatures. (**a**) Occurrence order of some pAD-biomarkers without specifying anatomical positions. (**b**) Occurrence order of atrophy with specified anatomical positions (ERC = Entorhical Cortex).

In addition, there are also studies that present evidence to the sequence of events for some biomarkers (Table 3). The major findings are described in Fig. 3, where the arrows indicate the sequence order. Fig. 3a reveals a macroscopic view regarding the occurrence order of several pAD-biomarkers, while the detailed order of regional changes in brain volume is presented in Fig. 3b.

## Discussion

The *PERD* offers a new methodology to study the mechanism of pAD by visualizing the relationship of the disease related factors through meta-analysis. It facilitates the detection of the primary causation of pAD and can be applied to the study of other diseases. Despite of the similarity of the proposed network with^4^ (in graph structure) and^5^ (in color coding the positive or negative correlation), *PERD* better utilizes color to illustrate the significance of correlation and width of edges to show the consistence in various trials on the same relationship. Due to inconsistence in study criteria for the correlation of pAD-biomarkers, edge-coding in *PERD* compromises to visualize the relationships. For example, the setting of the coefficients in the coding formula is empirically designed to reach an agreement with statistics concerning various studies. However, if researchers reach an agreement on imaging and correlation quantification methods, the evaluating potential of the proposed diagram will be highly improved. In addition, a map generated from *PERD* with time occurrences will also help to discover the areas where pathology of pAD is not well understood and to guide clinical studies to better understand the sequence of contributing factors.

A limitation of this work is that the current version of *PERD* only involves studies conducted within the preclinical phase of late-onset AD, the early onset AD or familial AD is not considered due to the difference in pathophysiology. In future work, the construction of *PERD* for familial pAD will help obtain a more comprehensive framework facilitating analysis of both types of pAD. While the current study mainly focuses on imaging biomarkers, other biomarkers of pAD could also be included. For example, episodic memory measurement was found to be sensitive to reflect subtle cognitive impairment (in amyloid-positive individuals), which presents in stage 3 of pAD^8^. Future converging evidence of neuropsychological variables for pAD staging could be included in *PERD* generation.

In addition, *PERD* could also become a platform to enable researchers who focus on pAD-biomarkers to communicate and update relevant findings through a website like *GenomeNet* (a Japanese network of database and computational services for genome research). With this type of platform, researchers may update relevant findings in a shared real time database of pAD-biomarkers. It would only be necessary to enter corresponding items as listed in the first row of Table 2. Embedded as a kennel tool in this website (*PERD-online*), *PERD* can be used to visualize synthesized results on corresponding correlations. Furthermore, authoritative pAD researchers on the leading edge would be also invited to regularly examine the operation of *PERD*-*online*, validate or exclude contributing factors as appropriate. Finally, joining efforts of researchers around the world concerning pAD will contribute to a constantly improving *PERD-online*, which enables better understanding of novel patterns from complex relationships as well as sufficient directions to channel further research efforts.

## Additional Information

**How to cite this article**: Shi, L. *et al.* Mapping the Relationship of Contributing Factors for Preclinical Alzheimer's Disease. *Sci. Rep.* **5**, 11259; doi: 10.1038/srep11259 (2015).

## Figures and Tables

**Table 1:** Parameterization for the relationship of pAD biomarkers.

**Factors**	**Description**
Report times(*T*)	The number of current studies regarding the same relationship.
Number of pAD subjects(*N*)	The number of subjects clinical presented with pAD (longitudinal study) or related to a high risk factor for pAD (cross-sectional study)
Correlation coefficient(*r*)	This coefficient denotes the degree of correlation between two biomarkers. The sign of r shows the correlation is positive (*sign(r) *=* *+1) or negative (*sign(r) = *−1).
Cross-sectional penalty(*α*)	If the study is cross-sectional, its result should bear a penalty, due to its restriction in observing the conversion in from NC (normal control) to real pAD patient. For cross-sectional study, *α* *=* 0.5; for longitudinal study, *α =* 1. The value of *0.5* in this penalty is determined empirically, and an alternative setting makes little difference with current findings in surveyed literatures.
Significance penalty(*β*)	This penalty is used to integrate the significance of a finding to width, where *β *=* (0.05/p)*^1^/2.We use *0.05* as the reference significance level and introduce the square operation to avoid extreme edge appearance.
Scaling coefficient(*A*)	This coefficient is empirically set to adjust the width of edge for best appearance. (*A *=* 0.01*)

**Table 2:** Summary of the literatures surveyed.

**Major biomarkers**	**Related factors**	**Imaging modality**	**Cross-sectional /longitudinal**	**Number of CN : pAD**	**Age: mean(SD/range) (CN : pAD)**	**Correlation coefficient (r)**	**p-value**	**Published date**	**Authors**
Aβ42(PET)	pAD^a^	PiB-PET	longitudinal	115:10	65.5(10.9):65.3(8.3)	0.141*	<0.05	2011–11	Vlassenko AG *et al.*^9^
Aβ42(PET)	Aβ42(CSF)	PiB-PET	cross-sectional	160:29	64.7(10.4)∇	−0.5123	<0.0001	2009–11	Fagan AM *et al.*^10^
Aβ42(CSF)	pAD^a^	NA#	longitudinal	28:7	85☆	−0.583	0.001	2003	Skoog I *et al.*^11^
CSF tau	Aβ42(PET)	PiB-PET	cross-sectional	160:29	64.7(10.4)∇	0.4508	<0.0001	2009–11	Fagan AM *et al.*^10^
CSF p-tau	Aβ42(PET)	PiB-PET	cross-sectional	160:29	64.7(10.4)∇	0.3856	<0.0001	2009–11	Fagan AM *et al.*^10^
CBF	pAD^a^	sMRI, PET	longitudinal	99:22	72.2(6.5):74.1(7.5)	0.43	0.04	2013–11	Beason Held LL *et al.*^12^
CBF(right superior medial frontal lobe)	tau	ASL	cross-sectional	24:8	76.6(8.4)∇	−1*	<0.001	2012–06	Stomrud E *et al.*^13^
CBF(right superior medial frontal lobe)	p-tau	ASL	cross-sectional	24:8	76.6(8.4)∇	−0.5*	<0.001	2012–06	Stomrud E *et al.*^13^
CBF(left frontal-temporal lobe)	p-tau	ASL	cross-sectional	24:8	76.6(8.4)∇	1*	<0.001	2012–06	Stomrud E *et al.*^13^
DMN	pAD^c^	rs-fMRI	cross-sectional	132:46	66.4(9.8):74.5(7.5)	−0.141*	<0.05	2014–04	Brier MR *et al.*^14^
DMN	Aβ42(CSF)	rs-fMRI	cross-sectional	136:71	70.8(6.3)∇	0.155	0.026	2013–10	Wang L *et al.*^15^
DMN	p-tau	rs-fMRI	cross-sectional	136:71	70.8(6.3)∇	−0.122	0.081^	2013–10	Wang L *et al.*^15^
Occipital network	pAD^c^	rs-fMRI	cross-sectional	132:46	66.4(9.8):74.5(7.5)	−0.141*	<0.05	2014–04	Brier MR *et al.*^14^
Modularity (graph metrics)	pAD^c^	rs-fMRI	cross-sectional	132:46	66.4(9.8):74.5(7.5)	−0.141*	<0.05	2014–04	Brier MR *et al.*^14^
FA(all the brain)	Aβ42(PET)	PiB-PET, DTI	longitudinal	59:27	59.7(6.0):60.0(6.0)	0.141*	<0.05	2014–02	Racine AM *et al.*^16^
FA(left fornix)	Aβ42(CSF)	PiB-PET, DTI	cross-sectional	11:9	76.2(5.8)∇	0.63	0.003	2014–05	Gold BT *et al.*^17^
MD(lateral frontal gray matter)	Aβ42(PET)	PiB-PET, DTI	longitudinal	59:27	59.7(6.0):60.0(6.0)	−0.392	0.024	2014–02	Racine AM *et al.*^16^
DR(left fornix)	Aβ42(CSF)	PiB-PET, DTI	cross-sectional	11:9	76.2(5.8)∇	−0.44	0.09^	2014–05	Gold BT *et al.*^17^
axD	Aβ42(CSF)	PiB-PET, DTI	cross-sectional	19:19	69.2(5.6):69.9(7.6)	0.57	0.013	2014–06	Molinuevo JL *et al.*^18^
Glucose metabolism	pAD^b^	FDG-PET	cross-sectional	36:21	76.1(5.9):77.7(5.5)	−0.333*	0.009	2014–09	Kljajevic V *et al.*^19^
Glucose metabolism	pAD^c^	FDG-PET	longitudinal	43:11	74.3(4.6):78.9(3.7)	−0.5*	0.004	2013–11	Ewers M *et al.*^20^
Glucose metabolism	pAD^b^	FDG-PET	cross-sectional	90:57	76(74~80):80(76~82)	−0.224*	0.02	2013–08	Knopman DS *et al.*^21^
LMB	Aβ42(PET)	SWI, PiB-PET	longitudinal	68:29	74.2(7.3)∇	0.141*	<0.05	2014–04	Yates PA *et al.*^22^
Atrophy(left amygdalo-hippocampal complex)	pAD^a^	sMRI	longitudinal	319:25	72.8(3.9):76.1(4.1)	1*	<0.001	2014–03	Bernard C *et al.*^23^
Atrophy(MTL)	pAD^b^	sMRI	longitudinal	90:57	76(74~80):80(76~82)	0.5*	0.004	2013–08	Knopman DS *et al.*^21^
Atrophy(MTL)	pAD^c^	sMRI	cross-sectional	136:71	70.8(6.3)∇	0.172	0.026	2013–10	Wang L *et al.*^15^
Atrophy(MTL)	pAD^a^	sMRI	longitudinal	25:8	71.2(4.0):71.5(2.1)	0.141*	<0.05	2011–04	Dickerson BC *et al.*^24^
Atrophy(MTL)	pAD^a^	sMRI	longitudinal	40:8	76.1(5.7):77.1(5.2)	0.141*	<0.05	2012–04	Tondelli M *et al.*^25^
Atrophy(parietal lobe)	pAD^a^	sMRI	longitudinal	35:9	69.1(7.7):73.8(4.3)	0.141*	<0.05	2011–03	Jacobs HI *et al.*^26^
Atrophy(superior frontal gyrus)	pAD^a^	sMRI	longitudinal	25:8	71.2(4.0):71.5(2.1)	0.141*	<0.05	2011–04	Dickerson BC *et al.*^24^
Atrophy(temporal pole)	pAD^a^	sMRI	longitudinal	25:8	71.2(4.0):71.5(2.1)	0.141*	<0.05	2011–04	Dickerson BC *et al.*^24^
Atrophy(posterior cingulate)	pAD^a^	sMRI	longitudinal	40:8	76.1(5.7):77.1(5.2)	0.141*	<0.05	2012-04	Tondelli M *et al.*^25^
Atrophy(basal forebrain)	Aβ42(PET)	sMRI, AV45-PET	cross-sectional	36:21	76.2(6.0):77.6(5.6)	0.45	0.04	2014–01	Grothe MJ *et al.*^27^
Atrophy(cerebral cortex)	Aβ42(CSF)	sMRI	cross-sectional	107:38	73.4(6.2)∇	1*	<0.001	2014–08	Fortea J *et al.*^28^
Atrophy(cerebral cortex)	Aβ42(CSF)	sMRI	cross-sectional	18:15	68.3(6.4):72.7(7.9)	NA(U-shaped)	<0.05	2011–07	Fortea J *et al.*^29^
Atrophy(cerebral cortex)	p-tau	sMRI	cross-sectional	107:38	73.4(6.2)∇	1*	<0.001	2014–08	Fortea J *et al.*^28^
Atrophy(cerebral cortex)	Aβ42(CSF) & p-tau	sMRI	cross-sectional	107:38	73.4(6.2)∇	−1*	<0.001	2014–08	Fortea J *et al.*^28^
Atrophy(whole brain)	pAD^b^	sMRI, PiB-PET	cross-sectional	148:75	78(75~83)∇	0.141*	<0.05	2014–05	Jack CR Jr *et al.*^30^

FA = Fractional Anisotropy; MD = mean diffusivity; DR = radial diffusivity; axD = axial diffusivity; LMB = lobar microbleed; CBF = cerebral blood flow; DMN = default mode network; sMRI = structural MRI.

^*^The correlation coefficients were estimated for the studies involving group comparisons or regression.

^#^Supplementary studies were used to connect the nodes of well-established biomarkers (Aβ) and pAD, although the publications were more than ten years ago.

^^^We included relatively significant findings with p-value larger than 0.05 but less than 0.1 to compute of the width of edge.

^a^The pAD subjects were diagnosed by neuropsychological assessment.

^b^The pAD subjects were defined by positive amyloid burden using PET.

^c^The pAD subjects were identified by CSF Aβ42 level.

^∇^These studies did not present age distributions for subjects in pAD group, so age information of overall subjects was listed instead.

^☆^Population-based study with focus on subjects with the same age.

**Table 3:** Literatures with findings on occurrence order of contributing factors of pAD.

**Inference of occurrence order of pAD-biomarkers**	**Published date**	**Authors**
Aβ42 metabolism and amyloid formation exceeds disruptions in CSF tau metabolism.	2009–11	Fagan AM *et al.*^10^
Early changes in CBF possibly present even earlier than amyloid-β accumulation.	2014–08	Wierenga CE *et al.*^31^
Hypometabolism exceeds atrophy in preclinical AD: amyloid load may affect synaptic activity, leading to synaptic loss and subsequent neuronal loss.	2014–09	Kljajevic V *et al.*^19^
Cortical thickening is associated with low CSF Aβ, followed by atrophy once CSF p-tau becomes abnormal.	2014–08	Fortea J *et al.*^28^
The atrophy change point in the ERC occurs first, indicating significant change 8–10 years prior to onset, followed by the hippocampus, 2–4 years prior to onset, followed by the amygdala, 3 years prior to onset.	2014–04	Younes L *et al.*^32^
